# Dabigatran reversal with idarucizumab in a patient undergoing heart transplantation: first European report

**DOI:** 10.1186/s12959-017-0147-z

**Published:** 2017-09-05

**Authors:** António Tralhão, Carlos Aguiar, Jorge Ferreira, Maria José Rebocho, Emília Santos, Dinis Martins, José Pedro Neves

**Affiliations:** 1 Department of Cardiology, Hospital de Santa Cruz, Centro Hospitalar de Lisboa Ocidental, Av. Professor Doutor. Reinaldo dos Santos, 2790-134 Carnaxide, Portugal; 2 Department of Cardiothoracic Surgery, Hospital de Santa Cruz, Centro Hospitalar de Lisboa Ocidental, Av. Professor Doutor. Reinaldo dos Santos, 2790-134 Carnaxide, Portugal; 3 Department of Cardiology, Hospital do Divino Espírito Santo, Av. D. Manuel I, 9500-370 Ponta Delgada, Portugal

**Keywords:** Dabigatran, Idarucizumab, Heart transplantation

## Abstract

**Background:**

Dabigatran is a direct thrombin inhibitor with a favorable effectiveness and safety profile when compared to vitamin K antagonists, both in randomized trials and real world registries of atrial fibrillation patients. Yet, physicians’ fear of high bleeding risk scenarios in daily clinical practice still precludes a more widespread use of oral anticoagulation. We hereby report a successful case of dabigatran reversal with the novel monoclonal antibody fragment idarucizumab in a patient undergoing heart transplantation.

**Case presentation:**

A 45-year old male patient on dabigatran for atrial fibrillation thromboprophylaxis was enlisted for heart transplantation due to end-stage ischemic heart failure. Upon donor availability and suitability and following the last intake of the drug 12 h previously, activated partial thromboplastin time was measured and found to be elevated. After general anesthesia and before extracorporeal circulation, idarucizumab was administered as two boluses of 2.5 g. Orthotopic heart transplantation ensued under full heparinization and cardiopulmonary bypass. Total chest tube output was 1125 mL after 3 days and 4 units of fresh frozen plasma and one platelet pool were administered in the operating room without further need for blood products. The post-operative period was uneventful.

**Conclusions:**

Idarucizumab was associated with an effective hemostasis in the setting of heart transplantation. Dabigatran may be considered as an alternative to vitamin K antagonists in heart transplant candidates with an indication for oral anticoagulation.

## Background

Atrial fibrillation (AF) is estimated to affect almost 6 million Europeans and the overwhelming majority will have an indication for oral anticoagulation [1]. Notably, oral anticoagulation is the most effective measure to reduce the risk of thromboembolic events and their devastating sequelae [2]. In recent years, stroke and systemic embolism prevention in AF patients has met a new era after the publication of the landmark trials of the non-vitamin K dependent oral anticoagulants (NOACs) [3–6]. Evidence from both randomized studies and real-world registries have consistently demonstrated at least an antithrombotic equipoise and less bleeding complications against vitamin K antagonists (VKA) [7, 8]. Another advantage shared by all NOACs is pharmacokinetic predictability, obviating the need for routine laboratory control and leading to greater patient comfort.

However, the lack of an antidote capable of restoring hemostasis in case of life-threatening bleeding or non-deferrable surgery after recent drug exposure still represented a major drawback for most of these agents [9]. In an effort to meet this unfulfilled need, a novel monoclonal antibody fragment was recently developed as an antidote for dabigatran, the first approved NOAC [10]. In a recent prospective study performed in patients with life threatening bleeding or the need for an urgent invasive procedure, idarucizumab promptly reverted the effect of dabigatran [11].

Nevertheless, each procedure bears its own bleeding risk and it is uncertain whether data from this small preliminary cohort study holds true in specific patient subsets. We hereby report a case of successful dabigatran reversal with idarucizumab in a patient undergoing heart transplantation.

## Case description

A 45-year-old male blood type A^+^ was enlisted for heart transplantation in December 2016 after entering stage D ischemic heart failure. His past history included a large anterior myocardial infarction (MI) in 2015 treated with alteplase and stent placement to the left anterior descending artery, abandoned smoking habits (60 pack-years), hypertension and bouts of self-terminating paroxysmal AF diagnosed during his various admissions for decompensated heart failure (left ventricular ejection fraction of 23%). Since he had a CHA_2_DS_2_-VASc of 3 [(Congestive heart failure, Hypertension, Age (doubled), Diabetes, Stroke/Transient ischemic attack/systemic thromboembolism (doubled), VAscular disease, Sex category), expected annual stroke rate of 3.2%], he was initially put on oral anticoagulation with rivaroxaban 20 mg (milligrams) once daily. His remaining medication was composed of carvedilol 6.25 mg bid (*bis in die*), ramipril 2.5 mg od (*omni die*), spironolactone 25 mg od, furosemide 40 mg bid, metolazone 10 mg od and atorvastatin 40 mg od. Laboratory evaluation showed a creatinine of 1.0 mg per deciliter (dL) [creatinine clearance of 119 milliliters (mL) per minute (min) by Cockroft-Gault’s formula] and a N-terminal-proB-type natriuretic peptide of 2566 picograms (pg) per mL, with no other relevant abnormalities. After being accepted for heart transplantation, he was given a priority of 6 out of 7, the highest for outpatients, 1–5 being reserved for inpatients in the waiting list. Following availability of idarucizumab at our institution, he was switched from rivaroxaban to dabigatran 110 mg bid, which was started 24 h (h) after the last dose of rivaroxaban.

On the 5th February 2017, a potential donor in brain death was deemed suitable for organ procurement. After a negative crossmatch and direct surgical inspection, the heart was excised at 11h45min and arrived at our institution, where the receiver was waiting in the operating room under general anesthesia. His laboratory workup 6 h previously showed a hemoglobin of 14.6 g per dL, platelet count of 104 000 per microliter, an activated partial thromboplastin time (aPTT) of 39.6 s (s) [1.35 times the mean reference value (reference range: 23–38 s)], a prothrombin time (PT) of 18.5 s (reference <14 s) and a fibrinogen of 4.2 g per liter (L) (reference range: 1.50–4 g per L). As the patient had taken the last dose of dabigatran in the night before (12 h previously) and both dilute thrombin time (dTT) and ecarin time (ET) were unavailable at the time at our institution, prompt restoration of hemostasis was considered necessary so that heart transplantation could be safely performed. After median sternotomy (16h15min) and pericardiotomy, cannulation of the aorta and both caval veins, idarucizumab was administered as two consecutive boluses of 2.5 g and the patient was cooled to 25° Celsius under nasopharyngeal monitoring. 50 mg of tranexamic acid were also administered intravenously, followed by a continuous infusion of 90 mg per hour during 2h30 min. First measured activated clotting time [(ACT),﻿ minutes after idarucizumab administration) was 147 s (reference range 80–160 s). After an intravenous bolus of 27 000 international units [(IU), 300 per kg of body weight] of non-fractionated heparin targeting an ACT between 400 and 480 s (together with priming of the extracorporeal circuit with 5000 IU), cardiopulmonary bypass (CPB) was started and orthotopic heart transplantation ensued. Peak ACT was >1000 s during CPB and 146 s after protamine (1 mg per 10 IU of heparin) and just before CPB exit. Hemoglobin was 14.5 g per dL pre-CBP and 11.9 g per dL post-CPB. Total blood products given during CBP amounted to 1 pool of platelets, 4 units of fresh frozen plasma and 1000 mL of recovered blood from the surgical field via Cellsaver®.

The patient was easily weaned off CPB and was transferred to the Intensive Care Unit (ICU) under dopamine [2 micrograms (μg) per kilogram (kg) per min] and isoproterenol (0.04 μg per kg per min). Cardiopulmonary bypass time was 120 min, aortic cross clamping time was 75 min and cold ischemia time was 165 min.

The following post-operative course was uneventful. Total pericardial and pleural drain yield was 300 mL by the 6th post-operative hour (baseline 300 mL in each drain after sternal closure) and total drainage was 1125 mL. No further blood products were administered.

## Discussion and conclusions

A normally functioning clotting system capable of contributing to an efficacious hemostasis is a fundamental prerequisite for performing major surgery. Understandably, quickly offsetting the effect of any anticoagulant would be desirable to minimize the occurrence of bleeding.

Dabigatran etexilate is a direct thrombin inhibitor, available in 75, 110 and 150 mg dosages in Europe and is currently approved for thromboprophylaxis in AF patients and in primary and secondary venous thromboembolism prophylaxis [12]. Peak plasma levels and full anticoagulant activity are attained following 2 h after the first drug intake, with a bioavailability of 3–7% after conversion to dabigatran and a 35% binding fraction to plasma proteins [13]. Metabolism occurs via the efflux transporter P-gp and the drug is not a substrate, inhibitor or inducer of CYP450 enzymes [13]. The drug undergoes predominant (80%) renal elimination [13]. Under normal kidney function, dabigatran half-life is 12–17 h but can reach 28 h in cases of severe renal impairment [13]. Unlike VKA, the level of anticoagulation cannot be quantitatively assessed by standard parameters such as TP or international normalized ratio, aPTT or ACT but can be achieved through other non-routinely performed tests such as dTT, which is extremely sensitive to dabigatran [14]. ET also shows a linear relation with increasing dabigatran concentrations and can be used alternatively [15]. In our center, both of these tests were unavailable. Nevertheless, given the pharmacokinetics of the drug and our patient’s estimated creatinine clearance, a relevant anticoagulant effect at the time of surgery was very likely, since a 12 h time interval corresponds to the drug’s steady-state trough level [16]. This may be further supported by an elevated aPTT (39.6 s or 1.35 times the mean reference value). Although the precise plasmatic level of dabigatran is not inferable from aPTT, the fact that it was prolonged is also considered to be a reliable surrogate for measurable drug levels [14]. Finally, and although documenting dabigatran anticoagulant activity seems reasonable, such strategy is not free of risks, as it may imply the notion that these tests are an indispensable pre-requisite to antidote utilization in everyday clinical practice. In accordance, idarucizumab’s summary of product characteristics does not mandate prior laboratory confirmation of anticoagulant activity [17].

As opposed to VKA, the lack of a fast-acting antidote capable of quickly restoring hemostasis in a case of major bleeding or urgent surgery was viewed by many as an Achilles heel that could hinder a more wider use of the drug when clinically appropriate. Due to dabigatran’s mechanism of action, usual antithrombotic effect reducing strategies such as concentrate of prothrombinic factors are less efficacious as thrombin remains inhibited [13]. Furthermore, alternative strategies such as removal by hemodialysis, although possible, are time consuming due to dabigatran’s large volume of distribution and not free of access-related vascular complications [18]. Idarucizumab is a humanized monoclonal antibody fragment with an affinity for dabigatran that is 350 times higher than that of thrombin, promptly neutralizing dabigatran [10]. In the RE-VERSE AD trial, 90 patients taking dabigatran (64% of which under the 110 mg dosage) and presenting with major blood loss (group A, *n* = 51) or requiring urgent surgery (group B, *n* = 39) received 5 g of idarucizumab as two 50 mL bolus given no more than 15 min apart. In group B, dTT and ET normalized in 93 and 88% of patients within minutes after the administration of idarucizumab [11].

Our case is among the first to report the reversal of dabigatran in the setting of urgent heart transplantation and the first, to the best of our knowledge, in a European country [19]. This patient would be excluded from the RE-LY trial due to foreseeable surgery in 3 months at the time of randomization [3]. Furthermore, in RE-VERSE-AD preliminary results, urgent surgery procedures did not include heart transplantation [11]. Notwithstanding, idarucizumab seems to constitute a useful, rapidly acting and effective dabigatran reversal agent in patients undergoing heart transplantation with recent drug exposure. The possibility of antagonizing dabigatran should be taken into consideration when weighing the risks and benefits of prescribing an oral anticoagulant to heart transplant candidates.

## Abbreviations

ACT: Activated clotting time﻿
AF: Atrial fibrillation
aPTT: Activated partial thromboplastin time
bid: *bis in die*
CHA_2_DS_2_-VASc: Congestive heart failure, Hypertension, Age (doubled), Diabetes, Stroke/Transient ischemic attack/systemic thromboembolism (doubled), VAscular disease, Sex category
CPB: Cardiopulmonary bypass
dL: Deciliter
dTT: Dilute thrombin time
ET: Ecarin time
g: Grams
ICU: Intensive care unit
IU: International units
Kg: Kilograms
L: Liter(s)
mg: Milligrams
MI: Myocardial infarction
min: Minute(s)
mL: Milliliter(s)
NOAC: Non-vitamin K dependent oral anticoagulants
od: *omni die*
pg: Picograms
PT: Prothrombin time
s: Seconds
VKA: Vitamin K antagonists
μg: Micrograms
